# Inhibition of ERK attenuates autophagy and potentiates tumour necrosis factor-α-induced cell death in MCF-7 cells

**DOI:** 10.1111/j.1582-4934.2008.00282.x

**Published:** 2008-02-08

**Authors:** U Sivaprasad, A Basu

**Affiliations:** Department of Molecular Biology and Immunology, University of North Texas Health Science Center and Institute for Cancer ResearchFort Worth, TX, USA

**Keywords:** TNF, autophagy, ERK 1/2, U0126

## Abstract

The role of autophagy in cell death is under considerable debate. The process of autophagy has been shown to lead to either cell survival or cell death depending on cell type and stimulus. In the present study, we determined the contribution of ERK1/2 signalling to autophagy and cell death induced by tumour necrosis factor-α (TNF) in MCF-7 breast cancer cells. Treatment of MCF-7 cells with TNF caused a time-dependent increase in ERK1/2 activity. There was an induction of autophagy and cleavage of caspase-7, -8, -9 and PARP. Pharmacological inhibition of ERK1/2 phosphorylation with U0126 or PD98059 resulted in a decrease in TNF-induced autophagy that was accompanied by an increase in cleavage of caspase-7, -8, -9 and PARP Furthermore, inhibition of ERK1/2 signalling resulted in decreased clonogenic capacity of MCF-7 cells. These data suggest that TNF-induces autophagy through ERK1/2 and that inhibition of autophagy increases cellular sensitivity to TNF.

## Introduction

Macroautophagy (referred to hereafter as autophagy) or ‘self-eating’ is a mechanism of degradation and recycling of a cell's own components, including long-lived proteins and damaged organelles [1]. Autophagy has a fundamental role in normal physiology as well as pathological conditions including cancer, infections and degenerative diseases like Alzheimer's [2]. During the process of autophagy, cytosolic constituents are sequestered by an isolation membrane that later encloses to form an autophagosome. The autophagosome fuses with a lysosome wherein enclosed materials are degraded by lysosomal enzymes [3, 4].

There is a considerable debate regarding the role of autophagy in cell death [2, 5, 6]. There is some pharmacologic and genetic evidence *in vitro* supporting a requirement for autophagy in the cell death response to nerve growth factor (NGF)-deprivation or cytosine arabinoside [7], serum and potassium deprivation [8], tumour necrosis factor-α (TNF) [9], caspase-8 inhibition [10], brevinin-2R [11], etoposide and staurosporine [12]. In contrast, autophagy can promote survival in stressful or nutrient-deprived conditions [1]. Furthermore, inhibition of autophagy was shown to increase susceptibility of cells to various stimuli including interleukin-3 (IL-3) deprivation [13], starvation [14] and infection [15]. In addition, inhibition of autophagy enhanced the cytotoxic effect of aloe emodin in U251 glioma, but not L929 fibrosarcoma cells [16]. Thus, autophagy has been associated with both cell death and survival depending on cellular context and stimulus.

Autophagy has been shown to precede apoptosis in human cervical cancer HeLa cells [17] and in *Drosophila* salivary glands during development [18, 19]. Furthermore, apoptosis and autophagy can occur simultaneously in the same cells [20], further complicating efforts to understand the contribution of autophagy to cell death. Thus, it is imperative to delineate the effect of autophagy induction and inhibition on cell death in a stimulus-specific manner.

Engagement of the receptor-mediated or extrinsic cell death pathway by ligands such as TNF and TNF-related apoptosis-inducing ligand (TRAIL) has been previously shown to induce autophagy. TRAIL induces autophagy in the lumen of MCF-10A cells grown in three-dimensional culture [21] and in prostate and mammary epithelial cells [22]. TNF induces autophagy in Ewing Sarcoma cells [23] and T-lymphoblastic cells [9]. The signalling mechanisms regulating TNF-induced autophagy, however, are not well defined. Activation of extracellular signal-regulated kinases (ERK)1/2 has been implicated in the induction of autophagy in response to several stimuli including amino acid deprivation [24], aurintricarboxylic acid [25], B-group soyasaponins [26] and curcumin [27]. While the role of the ERK pathway in TNF-mediated apoptosis is documented [28, 29] little is known about its involvement in TNF-induced autophagy. Furthermore, there are controversies whether the consequence of TNF-induced autophagy is to inhibit or potentiate cell death. Some reports implicate autophagy in TNF-induced cell death [9, 23] while others suggest that autophagy induction is a protective event against TNF-induced cytotoxicity [16]. Therefore, the aim of the present study was to determine the role ERK1/2 in TNF-induced autophagy and the contribution of autophagy to TNF-mediated cytotoxicity. We provide evidence that TNF induces autophagy *via* ERK1/2 and inhibition of ERK1/2 enhances sensitivity of MCF-7 human breast cancer cells to TNF-induced cell death.

## Materials and methods

### Materials

Human recombinant TNF was purchased from R&D systems (Minneapolis, MN, USA). Pharmacological inhibitors U0126 and PD98059 were purchased from EMD Biosciences (San Diego, CA, USA). Monoclonal antibodies to phospho-ERK1/2 and GAPDH, and polyclonal antibodies to enhanced green fluorescent protein (EGFP) and caspase-9 were purchased from Santa Cruz Biotechnology, Inc. (Santa Cruz, CA, USA). Monoclonal antibody to caspase-8 was from Biosource, Invitrogen (Carlsbad, CA, USA). Monoclonal antibodies to PARP, caspase-7 and ERK2 were purchased from BD Biosciences (San Diego, CA, USA). The rabbit polyclonal antibody against LC-3 was kindly provided by Dr. T. Yoshimori (Department of Cell Regulation, Research Institute for Microbial Diseases, Osaka University, Japan) [30]. Horseradish peroxidase-conjugated goat anti-mouse and donkey anti-rabbit antibodies were obtained from Jackson ImmunoResearch (West Grove, PA, USA). The enhanced chemiluminescence detection kit was from Amersham (Arlington Heights, IL, USA). The construct containing human LC3 tagged to EGFP was a kind gift from Dr. K. Kirkegaard (Department of Microbiology and Immunology, Stanford University, Stanford, CA, USA) and has been described previously [31].

### Cell culture and transfection

MCF-7 cells were maintained in RPMI 1640 medium supplemented with 10% heat-inactivated foetal bovine serum and 2 mM glutamine, and kept in a humidified incubator at 37°C with 95% air and 5% CO_2_. MCF-7 cells were transfected with EGFP vector alone or LC3-EGFP construct performed using Fugene HD (Roche; Indianapolis, IN, USA). Cells were selected in antibiotic (G418) to generate stable cell lines.

### Immunoblot analysis

Equivalent amounts of protein from total cellular extracts were electrophoresed by SDS-PAGE and transferred electrophoretically to poly(vinylidene difluoride) membrane. Immunoblot analyses were performed as described before [32].

### Clonogenic assay

Clonogenic assay was performed as described previously [33]. Briefly, MCF-7 cells were seeded in 60-mm tissue culture dishes and allowed to attach overnight. Cells were pre-treated with 0.01 and 0.1 μM U0126 for 1 h followed by treatment with or without indicated concentrations of TNF. Cells were then incubated in a humidified incubator at 37°C and 5% CO_2_ until there were at least 50 cells per colony in untreated cells. At the end of the incubation, cells were washed with phosphate-buffered saline (PBS) and incubated with a 0.25% crystal violet solution for 15 min. Following washes with PBS, colonies were counted using the colony-counting tool in the BioChemi System (UVP, Upland, CA, USA).

Data from clonogenic assays were analysed by anova followed by Tukey's *post hoc* test. All analyses were performed using the VassarStats web site program available at: http://faculty.vassar.edu/lowry/VassarStats.html. Differences were considered significant at *P***<** 0.05.

## Results

### TNF-induced PARP cleavage and LC3 processing in MCF-7 human breast cancer cells

**Fig. 1:**
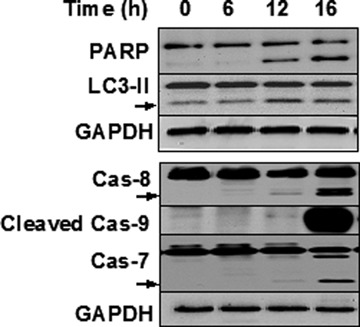
TNF induces apoptosis and autophagy in MCF-7 cells. Cells were treated with or without 0.1 nM TNF for the indicated time periods. Cell lysates were analysed by Western blotting performed with the indicated antibodies. The arrows indicate LC3-II form and processed forms of caspases. GAPDH was used to control for loading differences.

We have previously shown that TNF induces apoptosis in MCF-7 breast cancer cells [34, 35]. In the present study, we have examined if TNF influences autophagy in these cells. We monitored PARP cleavage that was shown to correspond to TRAIL- and TNF-induced apoptosis in MCF-7 cells [33, 36]. Treatment of MCF-7 cells with 0.1-nM TNF stimulated PARP cleavage in a time-dependent manner. The effect of TNF on PARP cleavage was observed at 12 hrs and was further increased at 16 hrs (Fig. 1). During apoptosis, inactive pro-caspases are cleaved to active processed forms. We therefore examined the effect of TNF on the processing of pro-caspases. As shown in Figure 1, TNF induced a time-dependent cleavage of initiator caspase-8 and -9, and effector caspase-7 to the processed forms.

Myosin-associated protein1-light chain 3B (LC3) is the mammalian ortholog of yeast Atg8 [30] and is intimately involved in the autophagy process. During autophagy, LC3 is conjugated to phosphatidylethanolamine (PE) creating a faster migrating band (LC3-II) that can be visualized in Western blots [30]. Thus, autophagy can be monitored by changes in the level of the LC3-II form. Autophagy is a ubiquitous cellular process and occurs at a basal level in almost all cells [2] although the extent varies with cell type. The level of LC3-II was readily visible in untreated MCF-7 cells (Fig. 1). TNF further stimulated LC3 processing, with maximal increases in LC3-II levels seen at 12 hrs (Fig. 1). The onset of both LC3 processing and PARP cleavage appeared to be concomitant.

### TNF stimulated formation of EGFP-LC3 puncta

**Fig. 2:**
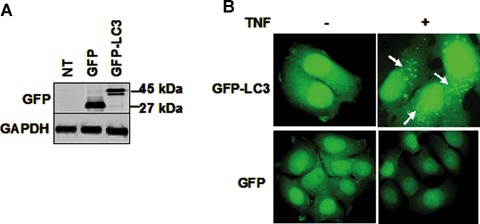
TNF induces formation of EGFP-LC3 puncta. (**A**) MCF-7 cells were stably transfected with EGFP vector alone or EGFP-LC3. Cell lysates were analysed by Western blotting performed with the indicated antibodies. GAPDH was used to control for loading differences. (**B**) MCF-7/EGFP and MCF-7/LC3 cells were treated with 0.1 nM TNF for 16 hrs. Formation of LC3 puncta was observed using a fluorescence microscope.

PE-conjugated LC3 is localized to autophagosomal membranes during autophagy [30]. Thus, redistribution of EGFP-tagged LC3 from diffuse pattern in the cytosol to distinct puncta in autophagic vesicles provides an alternative and widely accepted method to monitor autophagy [37]. We generated MCF-7 cells stably overex-pressing EGFP-LC3 (MCF-7/LC3) to assess localization of LC3 to autophagosomes (Fig. 2A). We found that TNF induced localization of LC3 to autophagosomes in MCF-7 cells overexpressing EGFP-LC3 as determined by the formation of punctate EGFP-LC3 (Fig. 2B).

### Inhibition of ERK1/2 decreased LC3-II levels and increased PARP cleavage in response to TNF

**Fig. 3:**
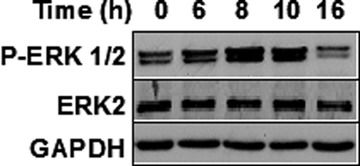
TNF activates ERK1/2. MCF-7 cells were treated with or without 0.1 nM TNF for the indicated time periods. Cell lysates were analysed by Western blotting performed with the indicated antibodies. GAPDH was used to control for loading differences.

Little is known about the signalling pathways that regulate TNF-induced autophagy in breast cancer. We found that TNF activated ERK1/2 in a time-dependent manner (Fig. 3) and was sustained up to 10 hrs following TNF treatment.

**Fig. 4:**
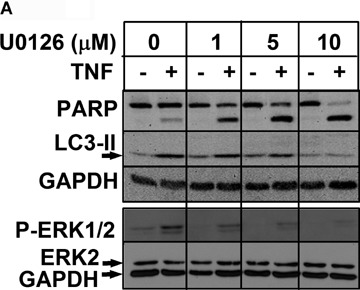

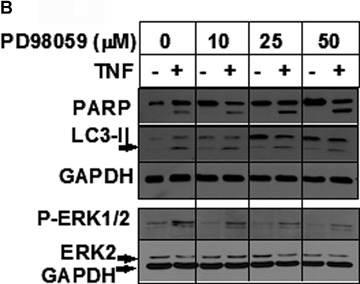
MAPK inhibitors decrease LC3-II levels and increase PARP cleavage induced by TNF. MCF-7 cells were pre-treated with indicated concentrations of U0126 (**A**) or PD98059 (**B**) for 1 h prior to treatment with or without TNF (0.1 nM) for 16 hrs. Cell lysates were analysed by Western blotting performed with the indicated antibodies. GAPDH was used to control for loading differences.

**Fig. 5:**
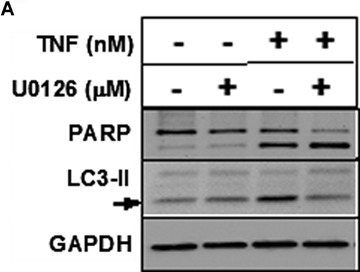

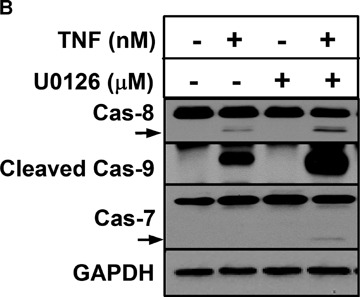

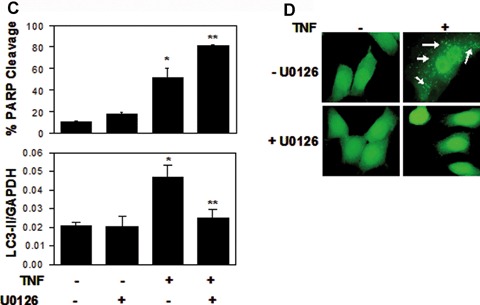
ERK inhibition attenuates TNF-induced autophagy. MCF-7 cells were pre-treated with 10 μM U0126 for 1 h prior to treatment with or without TNF (0.1 nM) for 16 hrs. (**A**) Cell lysates were analysed by Western blotting performed with the indicated antibodies. The arrow indicates the LC3-II form. (**B**) Arrows indicate processed forms of caspases. GAPDH was used to control for loading differences. (**C**) LC3-II levels and percentage PARP cleavage were determined by densitometric quantification of Western blots. Data are mean **±** S.E.M. of three separate experiments. * denotes significant difference from untreated controls (*P* < 0.05); ** denotes significant difference from TNF-treated samples (*P* <0.05) using Student's paired t-test. (**D**) MCF-7/EGFP and MCF-7/LC3 cells were treated with 0.1 nM TNF for 16 hrs. Formation of LC3 puncta was observed using a fluorescence microscope.

Signalling through the MAPK pathway has been associated with autophagy [24, 26, 38]. Therefore we determined the contribution of this pathway to TNF-induced autophagy. Pre-treatment of MCF-7 cells with the MEK1/2 inhibitors U0126 or PD98059 resulted in a concentration-dependent decrease in ERK1/2 activity as determined by phosphorylation of ERK1/2 (Fig. 4A and B). Decrease in ERK1/2 activity was associated with a decrease in LC3-II levels and a concomitant increase in the extent of cleavage of PARP, caspase-7, -8 and -9. (Figs. 4A and B and 5A and B). Densitometric quantification of Western blots revealed that TNF induced a fivefold increase in PARP cleavage compared to untreated controls. PARP cleavage was enhanced to eightfold when cells were pre-treated with U0126 (Fig. 5C). TNF induced a 2.5-fold increase in LC3-II levels that was completely abolished by pre-treatment with the MEK1/2 inhibitor (Fig. 5C). Similarly, U0126 pre-treatment resulted in a decrease in LC3 accumulation in autophagosomes as determined by the formation of EGFP-LC3 puncta (Fig. 5D). Thus, inhibition of ERK1/2 resulted in a decrease in autophagy and a concomitant increase in PARP and caspase cleavage.

### MEK1/2 inhibition sensitized cells to TNF-mediated decrease in clonogenic capacity

**Fig. 6:**
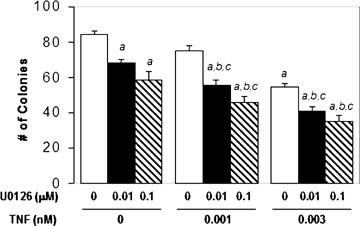
MAPK inhibition sensitizes cells to TNF-induced cell death. MCF-7 cells were pre-treated with indicated concentrations of U0126 for 1 h and then treated with or without TNF (0.001 or 0.003 nM). Clonogenic assay was performed as described in Materials and Methods. The bar graph represents average colonies + S.E.M. of three separate experiments. *a*, denotes significant difference from untreated control cells (*P* < 0.05); *b*, denotes significant difference from TNF-treated cells (*P* <0.05); *c*, denotes significant difference from U0126-treated cells (*P* < 0.05).

We further determined the consequence of decreased autophagy and increased PARP cleavage on long-term cell survival. In clonogenic assays (Fig. 6), we found that low concentrations of TNF (0.001 and 0.003 nM) had a modest effect on clonogenic capacity of MCF-7 cells. MEK1/2 inhibition alone decreased colony formation in a concentration-dependent manner. Treatment of cells with 0.01 and 0.1 μM U0126 caused a significant decrease in clonogenic capacity. In addition, pre-treatment with U0126 increased the ability of TNF to decrease clonogenic survival (Fig. 6). This suggests that activation of ERK1/2 induces autophagy in response to TNF as a protective mechanism against cell death.

## Discussion

In the present study we have shown that TNF can activate both apoptosis and autophagy in MCF-7 breast cancer cells. Pharmacological inhibition of ERK1/2 was associated with a decrease in TNF-induced autophagy while it augmented TNF-mediated apoptosis. Furthermore, the induction of autophagy by TNF *via* ERK1/2 signalling was a protective event since a decrease in autophagy increased PARP cleavage and decreased clonogenic capacity of the cells.

TNF induced LC3 processing as well as LC3 localization in autophagosomes in MCF-7 cells. Induction of autophagy by TNF has also been shown in lymphoblastic T cells [9], Ewing's sarcoma cells [23] and vascular smooth muscle cells [39]. In contrast, TNF alone did not stimulate autophagy in mouse C2C12 muscle cells [40] or in U251 glioma and L929 fibrosarcoma cells [16]. Furthermore, it has been shown that TNF could induce autophagy in MCF-7 cells only when NFκB signalling was absent [23]. We have shown, however, that TNF stimulates NFκB activity in MCF-7 cells [34]. Responses to TNF depend on several factors including cell type, concentration and time of exposure and this could explain the apparent discrepancies in the observations from various groups.

It has been proposed that cells with compromised apoptotic machinery undergo autophagy more readily than cells with intact apoptotic capacity [41–44]. In contrast, some reports suggest that cell death induced by autophagy is caspase-independent [45, 46]. MCF-7 cells do not express functional caspase-3, though they do express effector caspase-7 that exhibits some similarity in substrate specificities to caspase-3 [47]. It is, however, conceivable that cells expressing functional caspase-3 respond to TNF-induced apoptosis and autophagy differently.

Little is known about signalling pathways that regulate TNF-mediated autophagy. We have demonstrated that TNF induces autophagy in MCF-7 cells *via* the ERK1/2 pathway. The Ras-Raf-MAPK pathway, has emerged as a key regulator of autophagy [24, 26, 27, 48]. Sustained activation of ERK1/2 was associated with autophagy induction in H29 colon cancer cells [24]. In addition, increased ERK1/2 activity was necessary for B-group soyasaponin-induced autophagy [26]. Recently, Shinojima *et al.* (2007) showed that MEK inhibitors prevented curcumin-induced autophagy in glioma cells, suggesting that the activation of ERK is associated with the induction of autophagy [27]. In contrast, in C2C12 myotubes, autophagy was not associated with alterations in ERK1/2 phosphorylation [49].

There is some evidence that the JNK pathway can regulate autophagy in response to TNF [39] and through activated TRAIL receptor 2 [50]. TNF induced autophagy in vascular smooth muscle cells from atherosclerotic lesions by increasing the expression of MAP-LC3 mRNA *via* the JNK pathway [39]. However, we found that pre-treatment with a JNK inhibitor (SP600125) did not decrease autophagy in MCF-7 cells (data not shown) suggesting that this pathway may not be involved in up-regulating TNF-mediated autophagy.

Our results suggest that inhibition of signalling through ERK1/2 enhanced TNF-induced cell death and reduced clonogenic capacity. In contrast, there is evidence that autophagy contributes to apoptotic cell death. For example, inhibition of autophagy in T-lymphoblastic cells [9] and in Ewing's sarcoma cells [23] protected against TNF-induced death. Prins *et al.* (1998) found that cells that were more sensitive to TNF-induced cell death underwent greater extent of autophagy [51]. In addition, cells that were undergoing autophagy in response to starvation were more sensitive to TNF-induced apoptosis [40]. Our results are, however, in agreement with a recent report that MAPK inhibition enhanced curcumin-induced cytotoxicity in U87-MG and U373-MG glioma cells [27].

Whether autophagy enhances or diminishes the apoptotic response is a much-debated issue. Indeed, understanding of the interplay between autophagy and apoptosis is critical if the autophagy pathway is to be targeted effectively in the treatment of diseases. Taken together, data from the present study demonstrate that inhibition of ERK1/2 signalling results in decreased autophagy in response to TNF. This was associated with increased sensitivity of MCF-7 cells to TNF-induced cell death and further disruption of their clonogenic capacity. This suggests that TNF-induced autophagy is, in fact, protective against cytotoxicity in MCF-7 cells. Pharmacological inhibition of the MAPK pathway may therefore prove to be an attractive option to enhance drug efficacy in cancer cells where autophagy is a survival mechanism.
